# Endophthalmitis After Intravitreal Bevacizumab (Avastin) Injections: An Outbreak Investigated

**DOI:** 10.7759/cureus.36769

**Published:** 2023-03-27

**Authors:** Khawaja K Shoaib, Muhammad Awaid Abid, Saad Aziz

**Affiliations:** 1 Ophthalmology, Mughal Eye Hospital, Lahore, PAK; 2 Ophthalmology, Amna Inayat Medical College, Lahore, PAK

**Keywords:** intravitreal antibiotic injections, pseudomonas, ppv, pars plana vitrectomy, intravitreal (ivt) injection, endophthalmitis

## Abstract

Purpose

To investigate an endophthalmitis outbreak in different eye hospitals after intravitreal (IVT) bevacizumab (Avastin) injections over two days.

Place and duration of the study

Mughal Eye Hospital, Nov 2022 to Jan 2023.

Materials and methods

All cases reported with endophthalmitis in Mughal Eye Hospital after administration of anti-vascular endothelial growth factor IVT injections in different hospitals on November 8-9, 2022, were studied.

In all endophthalmitis cases, one to five IVT injections of antibiotics (vancomycin, ceftazidime, and dexamethasone) were given depending on the clinical picture.

Results

Thirty-six eyes of 34 patients who developed endophthalmitis were included in the study. Age (mean 53 years) ranged from 44 years to 70 years. There were 18 males and 16 females. Two patients had bilateral endophthalmitis. Thirty-three patients were given two to four IVT injections of antibiotics depending on the clinical response. Five cases of vitreous tap done before injecting antibiotics were found to have *Pseudomonas*. Anterior chamber wash was done in 15 cases to improve visualization for doing pars plana vitrectomy (PPV). In 24 patients PPV with silicon oil was done. Eighteen patients showed improvement as their hypopyon disappeared after IVT antibiotic injections. One patient developed corneal sloughing (she refused IVT antibiotic injections). One patient developed malignant/pupil block glaucoma. In two eyes, early PPV was done. Four eyes developed no perception of light. In our series, endophthalmitis patients started reporting one day after the injection. One patient who developed bilateral endophthalmitis developed bilateral cataracts with subluxations. One patient developed retinal detachment. One eye developed choroidal detachment.

Conclusion

IVT bevacizumab (Avastin) may be associated with an outbreak of endophthalmitis. *Pseudomonas* endophthalmitis is a very grave condition associated with severe morbidity.

## Introduction

Previously, cataract extraction was thought to be the most common surgical procedure, and endophthalmitis was considered to be the most dreadful complication. Intravitreal (IVT) injections are now the commonest procedure being done in ophthalmology. Their complications though uncommon are visually significant. Complications of IVT injections include inadvertent rupture of the lens, subconjunctival or vitreous hemorrhage, and retinal tears. The most important complication occurring after IVT injections is post-injection endophthalmitis. Endophthalmitis following bevacizumab injections (in-house compounded) had an incidence of 0.0377% in one cohort in Pakistan [1]. The patient developed pain, redness, and in most cases marked deterioration of vision (while pre-injection vision in many cases is not bad). This is in contrast to cataracts where a patient has already markedly decreased vision (preoperatively). Endophthalmitis if not treated timely and energetically frequently leads to a painful blind eye which requires evisceration. Prompt treatment however can save many eyes. In this study, we collected data about one cluster of endophthalmitis cases that occurred after IVT injections given over two days in different hospitals.

## Materials and methods

Anti-vascular endothelial growth factor (VEGF) IVT bevacizumab injections were administered in different hospitals in Lahore and Gujranwala (Punjab, Pakistan) on November 8-9, 2022. All the cases that subsequently developed endophthalmitis and were reported in Mughal Eye Hospital, Lahore (Punjab, Pakistan), were included in this study. anti-VEGF injections were administered in Mughal Eye Hospital, Al Mustafa Trust Eye Hospital, Ibrahim Eye Hospital in Lahore, and Muhammadi Eye Hospital in Gujranwala. In Mughal Eye Hospital, it is a standard routine to apply 10% povidone-iodine solution on the lid skin and 5% povidone-iodine solution in the conjunctival sac three minutes before the IVT injection. Inclusion criteria for the study included all cases of endophthalmitis reported in Mughal Eye Hospital (due to pain, redness, and decreased vision) whether initial anti-VEGF bevacizumab (Avastin) injections were given in Mughal Eye Hospital or in any other hospitals but referred to Mughal Eye Hospital when endophthalmitis developed. Most patients reported themselves the next day when they developed symptoms. Cases in which endophthalmitis developed following IVT bevacizumab (Avastin) but who did not report in Mughal Eye Hospital were excluded from the study.

All patients with endophthalmitis were given the following treatment for two weeks:

1. Systemic moxifloxacin, orally 400 mg every day for 14 days

2. Eye drops: moxifloxacin (Vigamox) for one hour, Deximox for four times/day, Atropine for three times/day, and tobramycin (Obrex Forte) for four to six times/day

3. IVT injections of antibiotics (vancomycin, ceftazidime, and dexamethasone) one to five times depending on the clinical response

4. Anterior chamber (AC) wash to improve visualization

5. Pars plana vitrectomy (PPV) if the eye did not respond to antibiotics

A PubMed search with “endophthalmitis after IVT anti-VEGF/Avastin injections” retrieved 167 results. Twelve articles were from 2022, 12 were from 2021, 10 were from 2020, and 9 were from 2019. All of these articles (a total of 53) were scanned.

## Results

**Figure 1:**
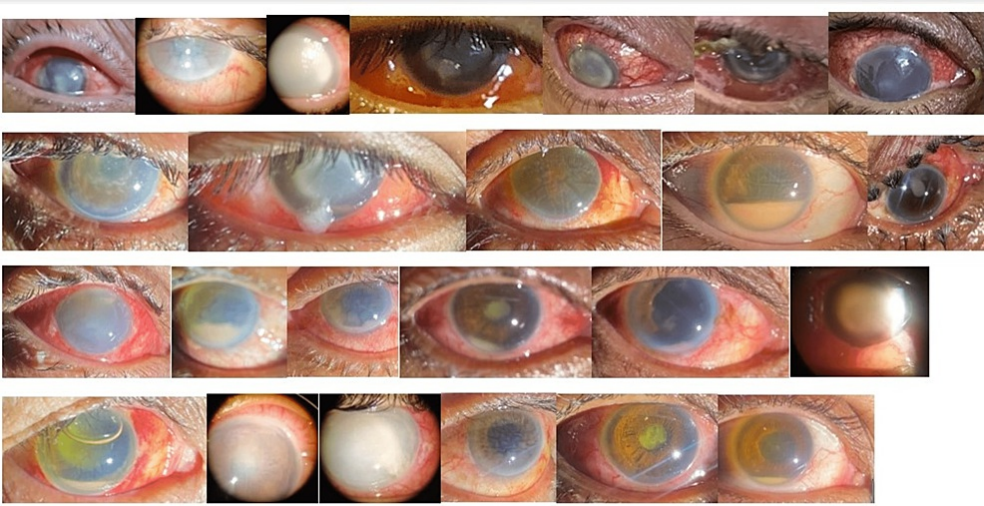
Endophthalmitis cases

Thirty-six eyes of 34 patients who developed endophthalmitis were included in this study (Figure 1).

Two patients had bilateral endophthalmitis. There were 18 males and 16 females. Age (mean 53 years) ranged from 44 years to 70 years. Thirty-three patients were given one to five (majority two to four) IVT injections of antibiotics depending on the clinical response (one patient refused IVT antibiotic injection). Roshe, Pakistan, supplied bevacizumab (Avastin) injections in filled syringes. They were contacted to investigate this happening. They claimed that they have laminar airflow, and anti-VEGF injections are prepared under strict aseptic conditions.

Five cases of vitreous tap done before injecting antibiotics were found to have *Pseudomonas*. AC wash was done in 15 cases to improve visualization for doing PPV. In 24 patients, PPV with silicon oil was done. Eighteen patients showed improvement, and their hypopyon disappeared after the first IVT antibiotic injection. In ten eyes of ten patients, the condition improved after the second IVT antibiotic injection, and pupillary dilatation started. One patient developed corneal sloughing (she refused IVT antibiotic injections). One patient developed malignant/pupil block glaucoma, and two underwent early PPV. Four eyes developed no perception of light (NPL). One patient developed pupillary block/malignant glaucoma. In our series, endophthalmitis patients started reporting one day after the injection. One patient who developed bilateral endophthalmitis developed bilateral cataracts with subluxations. One patient developed retinal detachment. One eye developed a choroidal detachment (Tables 1, 2).

**Table 1:** Signs in endophthalmitis IVT: intravitreal, NPL: no perception of light

Serial No.	Signs	No. of eyes
1	Hypopyon disappeared after the first IVT antibiotic injection and showed improvement	18
2	Corneal sloughing (she refused IVT antibiotic injection)	1
3	Malignant/pupil block glaucoma	2
4	NPL	4
5	Developed pupillary block/malignant glaucoma	1
6	Developed bilateral endophthalmitis and bilateral cataract with subluxations	2
7	Developed retinal detachment	1
8	Developed choroidal detachment	1

**Table 2:** Treatment done in endophthalmitis cases AC: anterior chamber, PPV: pars plana vitrectomy

No.	Treatment	No. of eyes
1	Vitreous tap before injecting antibiotics, found to have *Pseudomonas*	5
2	AC wash done to improve visualization	15
3	PPV with silicon oil was done	24

## Discussion

In our study, most of the IVT bevacizumab (Avastin) injections administered over two days resulted in endophthalmitis. In Mughal Eye Hospital, it is a standard routine to apply 10% povidone-iodine solution on the lid skin and 5 % povidone-iodine solution in the conjunctival sac three minutes before the IVT injection. Endophthalmitis happened in several hospitals in Lahore and Gujranwala. In all the cases, the supplier of the injections was the same (local distributor of the Roche company). It is likely that one lot/vial of the distributor got infected due to a poor dispensing environment. Though the distributor claimed that their laminar airflow and sterilization were satisfactory, that seemed to be the only weak link as the endophthalmitis occurred in many hospitals shortly after injections supplied by one pharmacy. Endophthalmitis cases following IVT bevacizumab can be encountered randomly, but more frequently these are found in clusters. While the incidence of endophthalmitis after IVT injections, in another study, was 0.028%, cultures were positive in 63 % of cases, and presenting visual acuity (VA) was the strongest factor associated with the final visual outcome [2]. The incidence of endophthalmitis was found to be 2.97 per 10,000 injections for IVT anti-VEGF injections. The endophthalmitis rate for bevacizumab was 3.64, for ranibizumab 1.39, and for aflibercept was 0.76 (per 10,000 injections). Endophthalmitis following bevacizumab injections (in-house compounded) had an incidence of 0.0377% in one cohort [3]. Strict preventive measures can dramatically reduce the infection rate. Out of 7,542 anti-VEGF injections, only one case of endophthalmitis occurred in Shifa International Hospital, Islamabad, Pakistan [4].

Talking during the procedure of IVT injection has especially been investigated for causing endophthalmitis as talking, sneezing, and coughing spread droplets from the respiratory tract of the administering doctor. In one study, cases of endophthalmitis in the “no-talking group” (0.0371%) were comparable with those occurring in the group in which physicians were wearing a face mask (0.0298%). Between the periods of 2009 to 2012 and 2016 to 2017, with a better understanding and improved prophylaxis, the incidence of endophthalmitis after IVT injection decreased, and the prognosis for vision improved. A "no-talking" policy during injections has decreased the endophthalmitis rate. Patients wearing a mask during anti-VEGF injection have not been found to decrease the endophthalmitis rate [5]. Like other intraocular procedures, adhesive face drape (e.g., Opsite) and (usually 5 %) povidone-iodine solution (and not scrub as it is toxic to the cornea) irrigation of conjunctival sac are important factors in the prophylaxis of endophthalmitis following IVT injections [6]. In our series, endophthalmitis occurred in spite of povidone-iodine solution prophylaxis in the conjunctival sac and the use of Opsite in 100% of cases in which IVT Avastin was administered. The syringe filling technique is an important consideration for IVT injection of anti-VEGF. Filling by a good professional pharmacy or manufacturer is better than self-drawing as for the incidence of endophthalmitis is concerned [7]. Dividing vial solution into syringes for IVT injections is safe if the compounding procedure is properly followed [8]. A large multicenter study in USA and Japan concluded that syringes that were prefilled were found to have a decreased endophthalmitis rate after IVT ranibizumab as compared to conventional preparations. In one study, endophthalmitis rates were found to be lower for bevacizumab and ranibizumab (vial and pre-filled) compared to aflibercept, dexamethasone implant, and triamcinolone. Triamcinolone was found to have a higher rate compared to all of the other medications [9]. As mentioned earlier, endophthalmitis usually occurs in clusters. Thus, it is possible that in some studies, the infection rate was less. In one series of IVT anti-VEGF injections for retinopathy of prematurity, no case of endophthalmitis was encountered. Many surgeons routinely advise antibiotic drops after the IVT injection. However, one study has concluded that topical antibiotics after IVT injections do not decrease the rate of endophthalmitis, but the use of steroid drops doubles the rate of endophthalmitis.

The occurrence of endophthalmitis depends on the patient’s immune status and the virulence/dose of the organisms. As far as the organisms responsible for endophthalmitis are concerned, one study found that *Staphylococcus *was the most commonly isolated organism [10]. In another study, it was found that *Streptococcus viridans* frequently cause aggressive but sometimes delayed endophthalmitis after IVT injections [11]. In a series of endophthalmitis after anti-VEGF, VA improved in the first six months with treatment and then remained at the same level over the next three to five years. In this series, 37% had VA that achieved the initial level (visual recovery group), whereas in 55% of patients' VA was worse than VA found initially (visual deterioration group). There was a difference in the organisms involved in the two groups. In the visual recovery group’s culture, coagulase-negative *Staphylococcus* was more, whereas, in the visual deterioration group, *Streptococcus* species, *Staphylococcus aureus*, and *Enterococcus faecalis* were more. In another series, there was 55.5% culture positivity for *Staphylococcus* species [3]. In our series, *Pseudomonas* was found in cases, and thus morbidity was high in our cases. Many times, intraocular pathology is bilateral. Injecting anti-VEGF in such cases is convenient for patients, doctors, and hospitals. However, in such cases, complications have grave effects. In one study, simultaneous bilateral injections were found to be safe [12], while in another study, bilateral IVT anti-VEGF injection resulted in bilateral endophthalmitis after two days [13]. In a series of 1418 bilateral same-day IVT anti-VEGF injections, no case of endophthalmitis was encountered [14]. In our series, two cases developed bilateral endophthalmitis which is truly a catastrophe for the patient. In one cohort of *Klebsiella* endophthalmitis following Avastin injection, prompt PPV saved many eyes [15]. Timing of endophthalmitis also depends on the patient’s immune status, virulence and dose of the organisms, and antibiotic post-injection treatment. In one study, the mean time between IVT bevacizumab injection and clinical endophthalmitis was 2.8 days (range one to six) [16]. In our series, patients with endophthalmitis started reporting one day after the injection. It reflects the high virulence of the organisms. Virulence generally depends on the replication time and the production of toxins. Pupillary dilatation in the ten eyes of ten patients was probably due to improved circulation in the iris. In ranibizumab injections, bacteria associated with the oral cavity were found in 27% of conventional culture-positive endophthalmitis cases (*Streptococcus viridans* in three cases, *Enterococcus faecalis* in three cases) compared to no case in the prefilled group [17].

In one series, acute bacterial endophthalmitis occurred in twenty-four patients following IVT injection of contaminated bevacizumab (single-dose syringes) in a single day. *Streptococcus *species were isolated in twenty-three cases (95.8%), and *Enterococcus* species were found in one case (4.2%) [18]. In our cases, severe ocular damage and visual deterioration not only reflected the high virulence of the organisms but probably also reflected some deficiencies in our treatment. IVT injections were given in some cases four or five times. This delayed PPV in many cases. Though the decision to proceed to PPV was taken independently (keeping in view only the deteriorating condition in spite of the IVT antibiotics), perhaps PPV should have been done earlier (instead of sticking to IVT injections).

## Conclusions

Endophthalmitis is a devastating complication occurring rarely after IVT anti-VEGF injections. However, many cases occurring in a cluster are really challenging, as the doctor has to not only promptly and energetically treat all the cases but also eradicate the source to prevent further happening. *Pseudomonas* endophthalmitis is a very grave condition associated with severe morbidity. In spite of IVT antibiotic injections and PPV, many eyes turn blind.
